# Carbamazepine regulates USP10 through miR-20a-5p to affect the deubiquitination of SKP2 and inhibit osteogenic differentiation

**DOI:** 10.1186/s13018-023-04169-7

**Published:** 2023-11-01

**Authors:** Huan Wang, Ziye Liu, Dawei Niu, Haobo Li, Yaguang Han, Jinhui Peng, Qirong Qian

**Affiliations:** 1 Department of Orthopedics, Shanghai Changzheng Hospital, No.415, Fengyang Road, Huangpu District, Shanghai, 200003 People’s Republic of China; 2 Department of Orthopedics, No. 971 Hospital of the PLA Navy, Qingdao, 266071 People’s Republic of China

**Keywords:** Carbamazepine, miR-20a-5p, USP10, SKP2, Osteoporosis

## Abstract

**Background:**

Antiepileptic drugs (AEDs) harm bone health and are significantly associated with osteoporosis development. In this study, we aimed to explore the mechanisms involved in carbamazepine (CBZ) and microRNA (miR)-20a-5p/ubiquitin-specific peptidase 10 (USP10)/S-phase kinase-associated protein 2 (SKP2) axis in osteoporosis.

**Methods:**

Human bone marrow mesenchymal stem cells (BMSCs) were treated with different concentrations of CBZ. Knocking down or overexpressing miR-20a-5p, USP10, and SKP2 cell lines were constructed. The expressions of miR-20a-5p, USP10, SKP2, runt-related transcription factor 2 (Runx2), Alkaline phosphatase (ALP), Osterix (Osx), osteocalcin (OCN) and Collagen I were detected with western blot (WB) and reverse transcriptase-quantitative polymerase chain reaction (RT-qPCR). Alizarin Red S (ARS) staining was performed to measure calcium deposition. Dual-luciferase assay and RNA immunoprecipitation (RIP) were applied to verify the binding relationship between miR-20a-5p and USP10. USP10 and SKP2 combination was verified by Co-Immunopurification (Co-IP). The stability of the SKP2 protein was verified by Cycloheximide chase assay.

**Results:**

CBZ could reduce cell activity. ALP activity and ARS staining were enhanced in the osteogenic induction (OM) group. The expressions of Runx2, ALP, Osx, OCN and Collagen I were increased. CBZ reduced miR-20a-5p expressions. Verification experiments showed miR-20a-5p could target USP10. USP10 increased SKP2 stability and promoted SKP2 expression. CBZ regulated miR-20a-5p/USP10/SPK2 and inhibited BMSCs osteogenic differentiation.

**Conclusions:**

CBZ regulated USP10 through miR-20a-5p to affect the deubiquitination of SKP2 and inhibit osteogenic differentiation, which provided a new idea for osteoporosis treatment.

**Supplementary Information:**

The online version contains supplementary material available at 10.1186/s13018-023-04169-7.

## Introduction

Osteoporosis is a bone disease characterized by decreased bone strength. It is a major public health problem that is becoming more prevalent as the world’s population ages [1]. Almost all postmenopausal women have osteoporosis [2, 3]. As a metabolic bone disease, it is characterized by a reduction in bone mass, bone quality, and microarchitectural deterioration [4]. Bone homeostasis relies on a delicate balance between bone resorption by osteoclasts and bone formation by osteoblasts. The imbalance of bone remodeling caused by osteoclast-mediated bone resorption and osteoblast-mediated bone formation results in this pathological bone disorder, namely osteoporosis [5, 6]. Therefore, current clinical research still focuses on how to improve osteogenesis or inhibit osteoclast differentiation.

Treatment with antiepileptic drugs (AEDs) contributes to achieving epileptic remission in approximately 70% of patients with epilepsy [7]. Typically, treatment with AEDs is long-term, and the discontinuation of AEDs induced by enzymes will increase the concentration of the inducing drug, and if the dose is not reduced at the same time, there will be a great risk of toxicity [8]. Studies have indicated that AEDs have detrimental effects on bone health and are significantly associated with the occurrence of osteoporosis [9]. Patients taking AEDs are at an elevated risk of developing osteoporosis and experiencing osteoporotic fractures [10]. Several mechanisms have been proposed for AEDs induced bone loss, such as vitamin D deficiency, hyperparathyroidism, estrogen deficiency, calcitonin deficiency, etc. [11, 12]. However, the specific molecular mechanism of its regulation is unknown and is worth further investigation.

New evidence has revealed that microRNAs play a crucial role in regulating osteoclastogenesis and bone resorption, influencing bone structure [13]. Specifically, miR-20a has been reported to promote osteogenic differentiation by upregulating the BMP/runt-related transcription factor 2 (Runx2) signaling [14]. Additionally, miR-20a has been found to promote osteogenesis by targeting bone morphogenetic protein and activin membrane-bound inhibitor (BAMBI) [15]. Zhang Z et al. demonstrated that diosgenin may exert anti-breakage effects against bone fracture in ovariectomized rats by upregulating miR-20a-5p expression in circulation and bone tissue [16]. These studies suggest that miR-20a-5p could promote osteogenic differentiation. However, the molecular mechanism of miR-20a-5p involvement in bone formation has not been reported. Studies have shown that silencing miR-20a-5p inhibited axonal growth and neuronal branching to repress epilepsy [17]. Based on this, we hypothesized that carbamazepine (CBZ), as a commonly used AEDs, might play a role by down-regulating miR-20a-5p.

S-phase kinase-associated protein 2 (SKP2) is an F-box subunit of SCFSkp2 ubiquitin ligase and a key regulator of G1/S transition [18]. Studies have demonstrated that circ_0087960 stabilized lysine demethylase 5B (KDM5B) by reducing SKP2-mediated ubiquitination degradation, thereby promoting osteogenic differentiation in periodontal ligament stem cells [19]. Furthermore, SKP2 has been found to inhibit osteogenesis by facilitating ubiquitin–proteasome degradation of Runx2 [20]. These reports suggested that SKP2 could inhibit osteogenic differentiation. Notably, Liao Y et al. reported that ubiquitin-specific peptidase 10 (USP10) could deubiquitinate SKP2 and promote SKP2 expression in chronic myeloid leukemia [21]. Interestingly, the Starbase prediction showed that miR-20a-5p can target USP10. However, the relationship between miR-20a-5p and USP10 has not been reported.

Based on the above background, we speculated that CBZ might alleviate the targeted inhibitory effect of miR-20a-5p on USP10 by inhibiting miR-20a-5p and promoting the deubiquitination and expression of SKP2, thereby inhibiting the osteogenic differentiation of human bone marrow mesenchymal stem cells (BMSCs). To this end, we conducted in vitro experiments to explore the effects of CBZ regulating miR-20a-5p on osteogenesis. This study provides new ideas and targets for the treatment of osteoporosis.

## Materials and methods

### Cell culture and treatment

BMSCs were purchased from the American Type Culture Collection (Fenghui Biotechnology, Hunan, China). The culture conditions were 89% high glucose DMEM (containing 584 mg/L L-glutamine and 110 mg/L sodium pyruvate) with 10% fetal bovine serum (FBS) and 100 U/mL penicillin/streptomycin. Cells were passaged during the logarithmic growth phase. The passage was performed by trypsin digestion. The 3-5th generation of BMSCs was used for further experiments. The cells were observed under a microscope and passaged when the cells were 90% fused. BMSCs were treated with various concentrations of CBZ (0, 10, 20, 40, 80, 160, 320 μM) for 24 h to detect the IC50 of CBZ (Additional file 1). Then, BMSCs were treated with CBZ at different concentrations (0, 10, 20, 40 μM) under the osteogenic induction medium (OM) (DMEM supplemented with 10% FBS, 0.1 μg/mL dexamethasone, 50 mg/L ascorbic acid and 10 mM β-Glycerol phosphate), replace the medium once in three days.

### Cell transfection

To overexpress or inhibit miR-20a-5p, BMSCs transfected with miR-20a-5p mimic, miR-20a-5p inhibitor, and the corresponding negative control (mimics-NC or inhibitor-NC). the above plasmids were obtained from GenePharma (Shanghai, China). USP10-expressing plasmid (pcDNA-USP10), the short hairpin RNA (shRNAs) targeting USP10 and their control empty vector were designed by Genepharma (Shanghai, China) and inserted into pGLV3/H1/EGFP vector. To generate target lentivirus for USP10 down-and upregulation, Lipofectamine 3000 (Thermo Fishier Scientific, USA) was applied to cotransfect the packaging vectors and lentiviral vectors according to the manufacturer’s instructions. Among them, 0.5 µg/mL miR-20a-5p mimic, USP10, miR-20a-5p inhibitor or sh-USP10 lentiviral vectors were used to transfect BMSCs for 48 h. After transfection, the cells were digested and inoculated into 96-well plates and cultured overnight in a 5% CO_2_ incubator at 37℃. On the second day, the cell density was 80%, cells were treated with OM containing CBZ.

### Reverse transcriptase-quantitative polymerase chain reaction (RT-qPCR)

After observing BMSCs under a microscope, RT-qPCR experiments were performed once the cell fusion in the six-well plate reached 90%. The expression levels of miR-20a-5p, USP10, runt-related transcription factor 2 (Runx2), Alkaline phosphatase (ALP), Osterix (Osx), osteocalcin (OCN), and Collagen I were analyzed using RT-qPCR. Total RNA was extracted by the Trizol method. cDNA reverse transcription Kit (#CW2569, Beijing ComWin Biotech, China) was applied to reverse transcription RNA into cDNAs by applying 5 μg/mL RNA. Ultra SYBR Mixture (#CW2601, Beijing ComWin Biotech, China) was utilized to measure the relative expressions of the genes on the fluorescence quantitative PCR (QuantStudio1, Thermo, USA). cDNA was denatured at 95 ℃ for 10 min, and then annealed at 60 ℃ for 30 s, finally, it extended at 70 ℃ for 1 min, 40 cycles in total GAPDH or U6 was used as the reference gene, and the relative level of genes was calculated by the 2 ^−ΔΔCt^ method. The primer sequences were listed as follows (5’-3’):

- miR-20a-5p-F: 5’- UAAAGUGCUUAUAGUGCAGGUAG -3’,
- miR-20a-5p-R: 5’- CUACCUGCACUAUAAGCACUUUA -3’;
- U6-F:5’- CTCGCTTCGGCAGCACATATACT -3’,
- U6-R: 5’-CGCTTCACGAATTTGCGTGT -3’;
- USP10-F: 5’-TTATGAGAAGACTGGTGGGT-3’,
- USP10-R: 5’-TGTTGCCGTGATGGTAGA-3’;
- SKP2-F: 5’-AGTCTCTATGGCAGACCTTAGACC-3’,
- SKP2-R: 5’-TTTCTGGAGATTCTTTCTGTAGCC-3’;
- GAPDH-F: 5’-GGAGCGAGATCCCTCCAAAAT-3’,
- GAPDH-R: 5’-GGCTGTTGTCATACTTCTCATGG-3’.

### Western blot (WB)

After observing BMSCs under a microscope, WB experiments were carried out once the cell fusion in the six-well plate had reached 90%. According to instructions, total proteins were extracted from cells and tissues using RIPA lysate buffer (#P0013B, Beyotime Biotechnology), and proteins were quantified using the BCA protein Assay Kit (KeyGen, Nanjing, China). The mixed SDS-PAGE loading buffer (#MB2479, Meilunbio) was heated in a boiling water bath at 100℃ for 5 min. The proteins were transferred to a PVDF membrane (Millipore, MA, USA). According to the results of protein quantification, the sample was added. First 80 V and then 100 V until the bromophenol blue reaches the bottom of the gel. Then the membrane was transferred, the gel was cut respectively according to the molecular weight, and the PVDF film of the corresponding size was cut. When the membrane was transferred, the constant current was 300 mA. And membranes were incubated with antibodies against USP10 (AB70895, 1:2000, Abcam), SKP2 (AB214666, 1:1000, Abcam), and Runx2 (AB236639, 1: 1000, Abcam), ALP (AB229126, 1:1000, Abcam), Osx (AB209484, 1:1000, Abcam), OCN (AB133612, 1: 1000, Abcam), Collagen I (AB34710, 1:1000, Abcam) and GAPDH (10,444-1-AP, 1:500, Proteintech) overnight. Then HRP Goat Anti-Mouse IgG (SA00001-2, 1:5000, Proteintech) or HRP Goat Anti-Rabbit IgG (SA00001-2, 1: 6000, Proteintech) was incubated for 60 min. The membrane was incubated with ECL Western Blotting Substrate (Solarbio biotech) for 1 min. Protein bands’ chemiluminescence signals were captured using the Odyssey Infrared Imaging System (Li-Cor Biosciences, Lincoln, NE, USA) with GAPDH as a reference gene.

### MTT

MTT was applied to characterize the cell viability. The cells in the logarithmic growth phase were digested by 0.25% trypsin counted, and inoculated into a 96-well plate at a density of 5 × 10^3^ cells/well, 100 μL per well, and cultured for 24 h. 10 μL MTT was added to each well and incubated with cells at 37℃ with 5% CO_2_ for another 4 h, and then 150 μL DMSO was added to each well. After shaking slowly for 10 min at room temperature, absorbance at 490 nm was analyzed by Bio-Tek microplate (MB-530, Heales, China).

### ALP staining

To assess the effects of CBZ on ALP activity, BMSCs were seeded into 6-well culture plates at a density of 2 × 10^5^ cells/well, and incubated with 2 mL/well OM containing different concentrations of CBZ (0, 10, 20, 40 μM) CZB for 14 days to induce differentiation. After inducting 14 days, the cell culture medium was removed and the cells were washed with PBS 2–3 times. Cells were fixed in 4% formaldehyde for 30 min, after which they were washed three times with PBS and stained by Alkaline Phosphatase Kit (MAK447, Sigma-Aldrich) under the guidance of the instructions. Then cells were observed under a microscope.

### Alizarin Red S (ARS) staining

BMSCs were inoculated in 6-well plates with 2 × 10^5^ cells/well density, cultured with OM containing CBZ for 14 days, and the medium was changed every three days. After inducting 14 days, the cells were fixed with 4% paraformaldehyde for 30 min. The neutral formaldehyde solution was absorbed and rinsed twice with 1 × PBS. Then 1 mL ARS (PH 4.2; Sigma-Aldrich) was added for 3–5 min. Alizarin red dye was absorbed and rinsed with 1 × PBS 2–3 times. The culture plates were placed under a microscope to observe the staining effect of osteogenesis.

### Bioinformatics prediction and dual-luciferase reporter assay

Starbase (http://starbase.sysu.edu.cn/) was applied to predict the binding sites between USP10 and miR-20a-5p. The cells were placed in a 24-well plate with a density of 5 × 10^4^ cells per well. Wild-type (WT) or mutant (MUT) USP10 fragments were constructed and inserted into the pmirGLO vector (Promega). According to the instructions, the recombinant vector was transfected into microglia cells using lipofectamine 3000 reagent (Thermo Fisher Scientific), mimic NC and miR-20a-5p mimic (50 nM) were simultaneously transferred into the cells. Finally, the luciferase activity was assessed by Nano-Glo dual luciferase reporting method (Promega).

### RNA immunoprecipitation (RIP) assay

According to the manufacturer's instructions, the combination of miR-20a-5p and USP10 was validated using the EZMagna RIP kit (Millipore, Massachusetts, USA). Briefly, the BMSCs were dissolved in RIP lysate buffer at 4 °C for 30 min and then incubated with RIP buffer containing magnetic beads. These beads were conjugated against Ago2 (CST, Boston, USA) and anti-Rabbit IgG (Negative Control, CST, Boston, USA), respectively. The precipitated RNA was analyzed using RT-qPCR technology, with total RNA as input controls.

### Co-Immunopurification (Co-IP)

The interaction between USP10 and KLF4 was detected by Co-IP. BMSCs were lysed using Co-IP lysate to extract proteins. Protein supernatants were incubated with Normal rabbit IgG or USP10 (AB70895, Abcam), and SKP2 (AB214666, Abcam) antibodies at 4℃ overnight with rotation. The Co-IP lysate was then added to Protein A/G agarose beads, mixed, and centrifuged to collect the precipitate. Following antibody incubation, the cell lysate was fully incubated with agarose beads for 2 h at 4℃ to allow antibody coupling to the beads. The agarose beads were washed using Co-IP lysate buffer, and WB subsequently analyzed the coupling product.

### Cycloheximide chase assay

As mentioned above [22], the effect of USP10 knockdown on the stability of SKP2 protein was detected by cycloheximide chase assay. sh-USP10 plasmid was transfected into the cells, 24 h after transfection, cycloheximide (50 μg/mL, Sigma) was used to treat cells for 0, 3, 6, 9, 12 h. The lysate was prepared, and the WB experiment was performed as described above.

### Statistical analysis

Graphpad 8.0 was used for statistical analysis, and the three independent experimental data were expressed as mean ± standard error, which was repeated at least three times. One-way analysis of variance (ANOVA) was applied for comparison between multiple groups. The *p*-value lower than 0.05 was considered statistically significant.

## Results

### CBZ inhibited cell activity and osteogenic differentiation of BMSCs

**Fig. 1:**
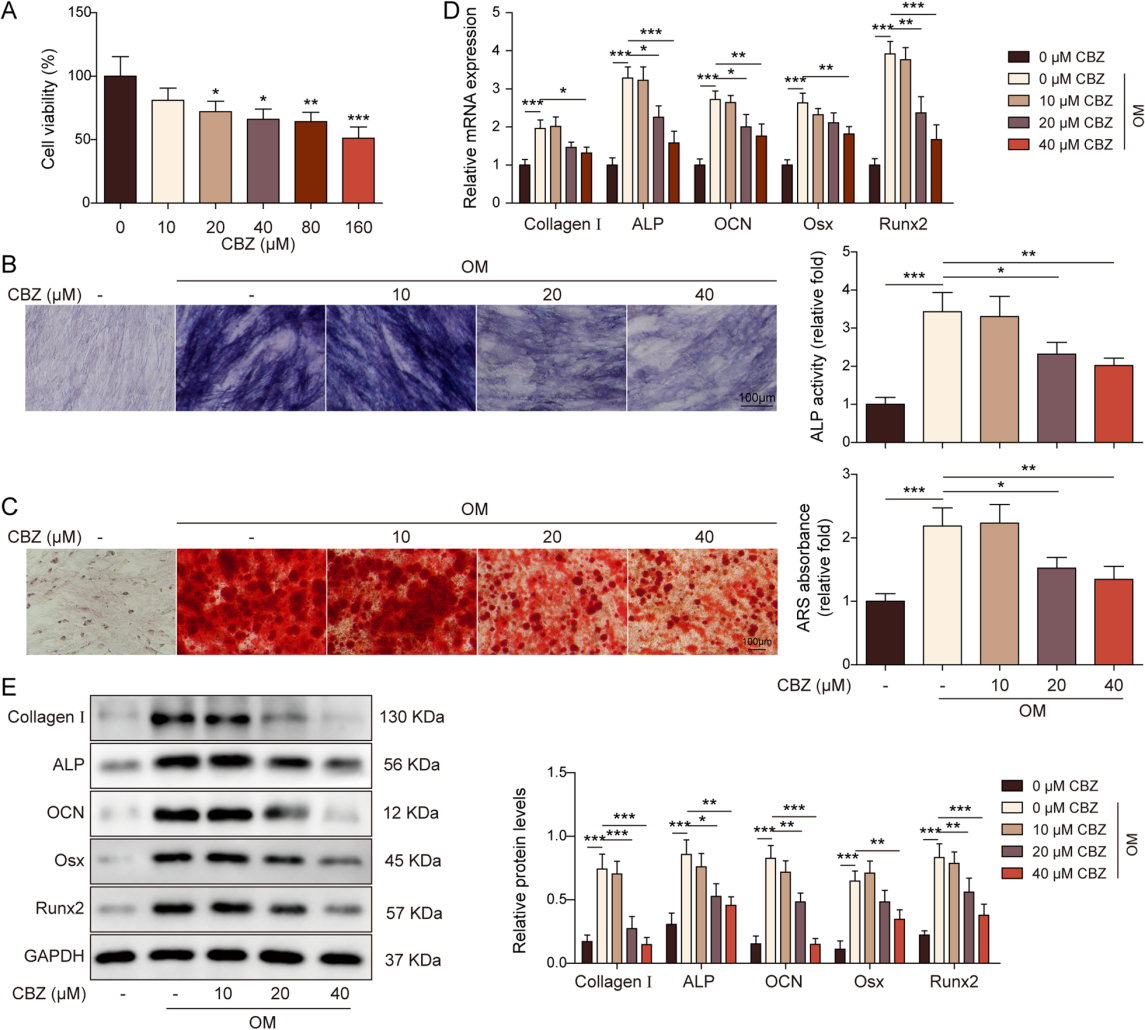
Carbamazepine inhibited cell activity and osteogenic differentiation of human bone marrow mesenchymal stem cells. **A** BMSCs were treated with CBZ, cell activity was detected by MTT. ALP activity (**B**) detection and ARS staining (**C**) were used to check osteogenic differentiation ability. Scale bar = 100 µm. The expression of Runx2, ALP, Osx, OCN and Collagen I was detected by RT-qPCR (**D**) and WB (**E**). The measurement data were presented as mean ± standard error. All data were obtained from at least three replicate experiments. *p < 0.05, ** *p* < 0.01, ***p < 0.001

To investigate the effect of CBZ on osteogenic differentiation, BMSCs were treated with various concentrations of CBZ (0, 10, 20, 40, 80, 160 μM) for 24 h. 10 μM CBZ did not affect cell activity within 24 h, while 20–160 μM CBZ could decrease cell activity (Fig. 1A). Then, BMSCs were treated with (0, 10, 20, 40 μM) CBZ and osteogenic induction medium OM for subsequent experiments. Compared with the blank control group, ALP activity and ARS staining were enhanced in the OM group, indicating successful osteogenesis. BMSCs treated with CBZ showed decreased ALP activity, ARS staining and mineralization deposition, suggesting that CBZ could inhibit osteogenesis (Fig. 1B-C). As shown in Fig. 1D-E, the mRNA and protein expressions of Runx2, ALP, Osx, OCN and Collagen I were increased in the osteogenic medium. While these indicators decreased after incubation with CBZ (20–40 μM).

### MiR-20a-5p alleviated the inhibition of osteogenic differentiation induced by CBZ

**Fig. 2:**
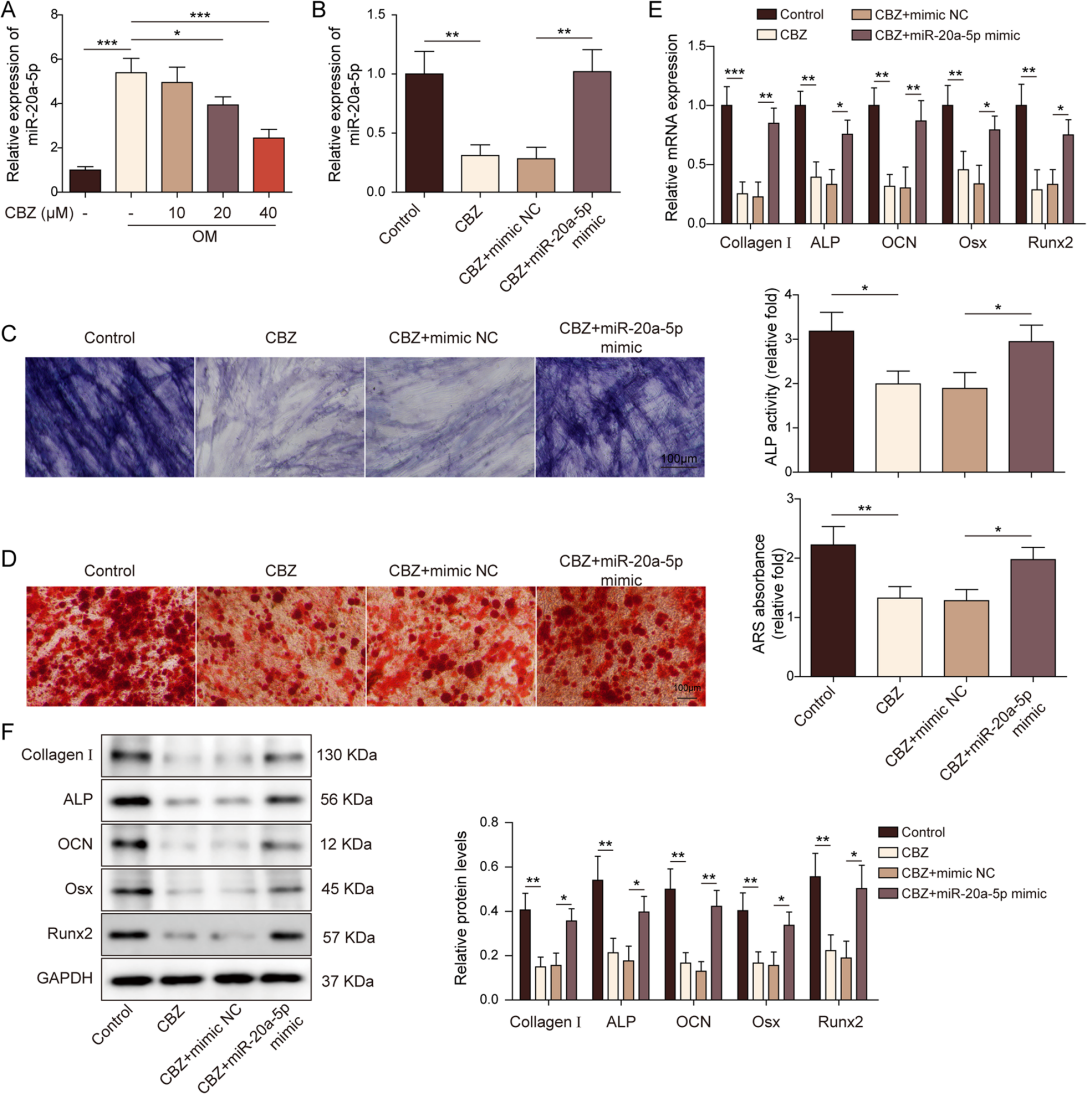
microRNA-20a-5p alleviated the inhibition of osteogenic differentiation induced by carbamazepine. **A**–**B** The miR-20a-5p level was detected by RT-qPCR. **C** ALP activity detection. **D**. ARS staining was used to detect calcium deposits. Scale bar = 100 µm. RT-qPCR (**E**) and WB (**F**) were used to detect the expression of Runx2, ALP, Osx, OCN and Collagen I. The measurement data were presented as mean ± standard error. All data were obtained from at least three replicate experiments. *p < 0.05, ** *p* < 0.01, ***p < 0.001

Firstly, constructing stable overexpression BMSCs of miR-20a-5p, and then BMSCs were treated with different concentrations (0, 10, 20, 40 μM) of CBZ under OM. BMSCs were grouped into the following groups: control, CBZ, CBZ + mimic NC, and CBZ + miR-20a-5p mimic groups. As demonstrated in Fig. 2A-B, osteogenic medium promoted miR-20a-5p expression, while significantly decreased by CBZ. miR-20a-5p mimic relieved the inhibitory effect of CBZ on miR-20a-5p. In addition, transfection of miR-20a-5p mimic reversed the decrease of ALP activity and calcium deposition in CBZ-stimulated BMSCs (Fig. 2C-D). This suggested that overexpression of miR-20a-5p could alleviate CBZ induced osteogenic differentiation. RT-qPCR and WB subsequently revealed that miR-20a-5p increased the mRNA and protein expressions of Runx2, ALP, Osx, OCN and Collagen I in OM-treated BMSCs and could reverse regulatory effect of CBZ on these osteogenic differentiation-related proteins (Fig. 2E-F).

### miR-20a-5p directly targeted USP10

**Fig. 3:**
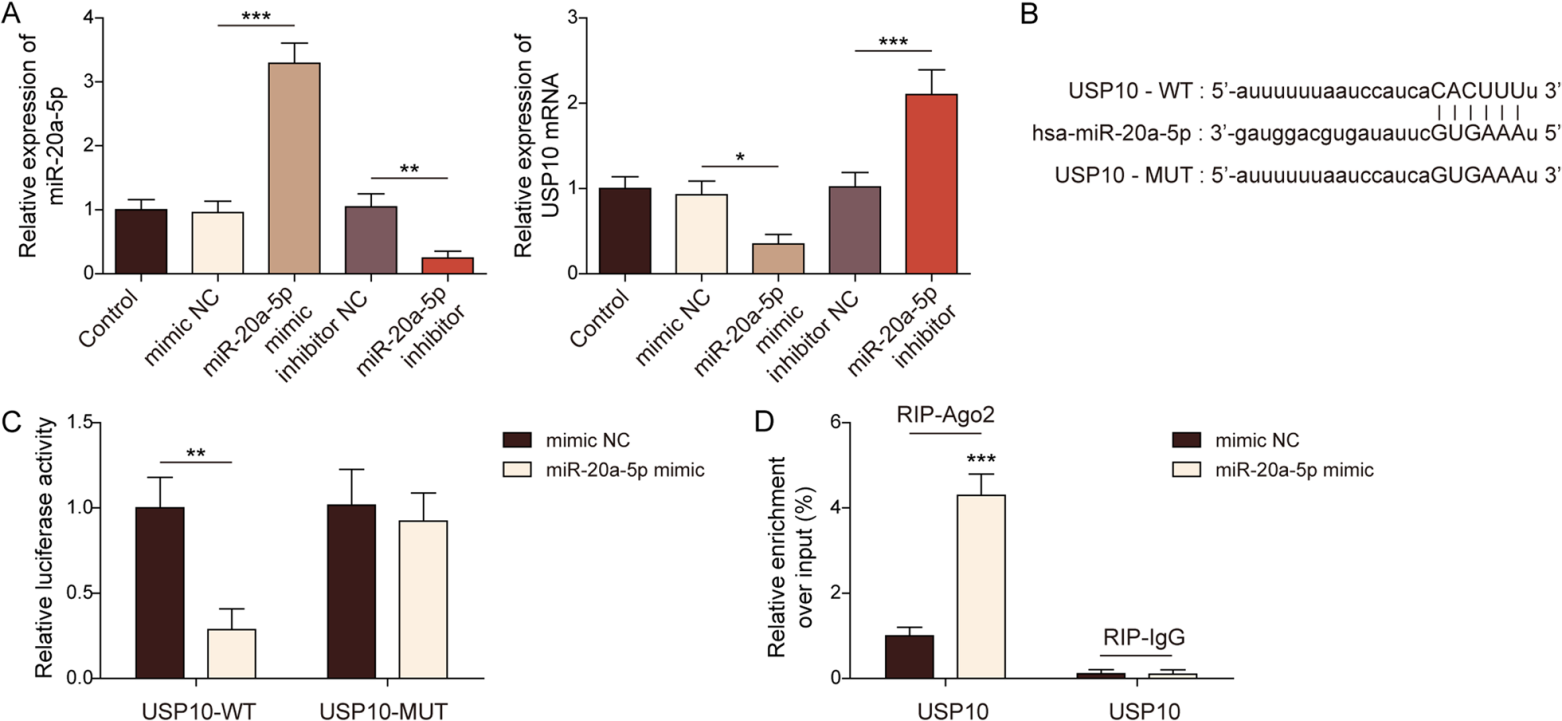
microRNA-20a-5p directly targeted ubiquitin-specific peptidase 10. **A** The levels of miR-20a-5p and USP10 were detected by RT-qPCR. **B** Prediction of binding sites between miR-20a-5p and USP10. **C** Dual-luciferase report assay detected the binding relationship between miR-20a-5p and USP10. **D** RIP verified the binding relationship between miR-20a-5p and USP10. The measurement data were presented as mean ± standard error. All data were obtained from at least three replicate experiments. *p < 0.05, ** *p* < 0.01, ***p < 0.001

Then miR-20a-5p mimic and/or inhibitor and their negative control were transfected to BMSCs to establish the miR-20a-5p mimic and/or inhibitor cell model (Fig. 3A). We subsequently studied the mechanism of miR-20a-5p in regulating osteogenic differentiation on BMSCs. As shown in Fig. 3B, the Starbase database predicted that miR-20a-5p posed the potential binding sites on USP10. And the results of RT-qPCR evidenced that miR-20a-5p targeted USP10 and repressed its expression (Fig. 3A). Dual-luciferase reporter gene assay confirmed that miR-20a-5p mimic significantly decreased the fluorescence intensity of WT-USP10 (Fig. 3C). Furthermore, the RIP analysis also validated that miR-20a -5p had potential binding sites with USP10, and the relative enrichment of USP10 was significantly higher in the anti-Ago2 groups than the anti-IgG groups (Fig. 3D).

### Knockdown of USP10 blocked the osteogenic inhibition induced by CBZ

**Fig. 4:**
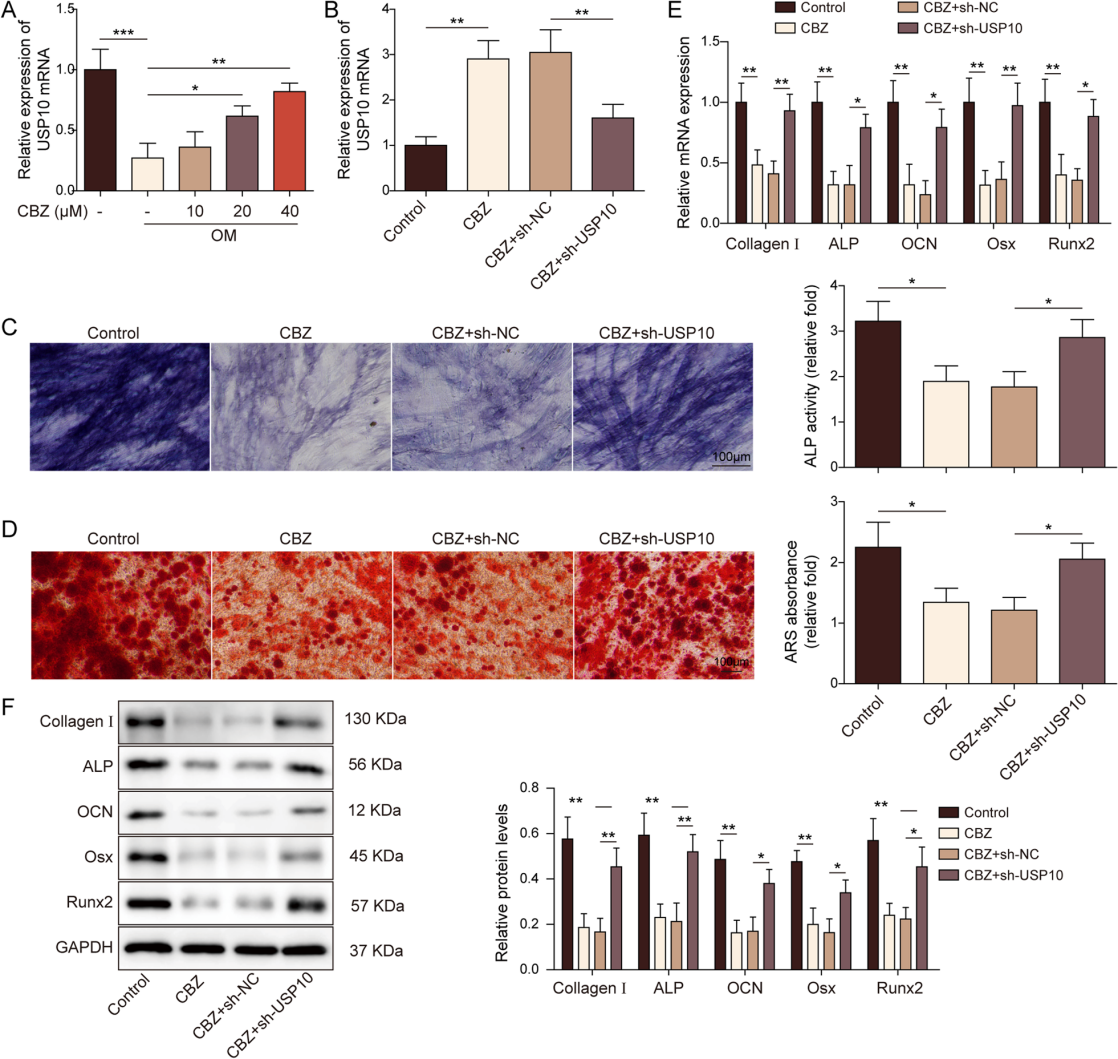
Knockdown of ubiquitin-specific peptidase 10 blocked the osteogenic inhibition induced by carbamazepine. **A** The levels of USP10 were detected by RT-qPCR. **B** USP10 level was detected by RT-qPCR. ALP activity (**C**) detection and ARS staining (**D**) were used to check osteogenic differentiation ability. Scale bar = 100 µm. RT-qPCR (**E**) and WB (**F**) were used to detect the expression of Runx2, ALP, Osx, OCN and Collagen I. The measurement data were presented as mean ± standard error. All data were obtained from at least three replicate experiments. *p < 0.05, ** *p* < 0.01, ***p < 0.001

Next, we investigated the function of USP10 in CBZ-mediated inhibition biological effects on osteogenic differentiation, and cells were administrated with CBZ, and OM or sh-USP10. OM could reduce USP10 level. However, after adding CBZ to BMSCs, the level of USP10 increased in a concentration-dependent manner (Fig. 4A). As shown in Fig. 4B, BMSCs transfected with 5 µL sh-USP10 reversed the positive regulation of 40 μM CBZ on USP10. Moreover, after 40 μM CBZ treatment, the ALP activity and ARS staining intensity of cells decreased, while the silencing of USP10 increased them (Fig. 4C-D). In line with the above results, RT-qPCR and WB indicated that compared with the control group, the mRNA and protein expressions of Runx2, ALP, Osx, OCN and Collagen I in BMSCs treated with CBZ decreased, and transfection of sh-USP10 elevated Runx2, ALP, Osx, OCN and Collagen I mRNA and protein expressions on BMSCs (Fig. 4E-F).

### USP10 increased SKP2 stability and promoted SKP2 expression

**Fig. 5:**
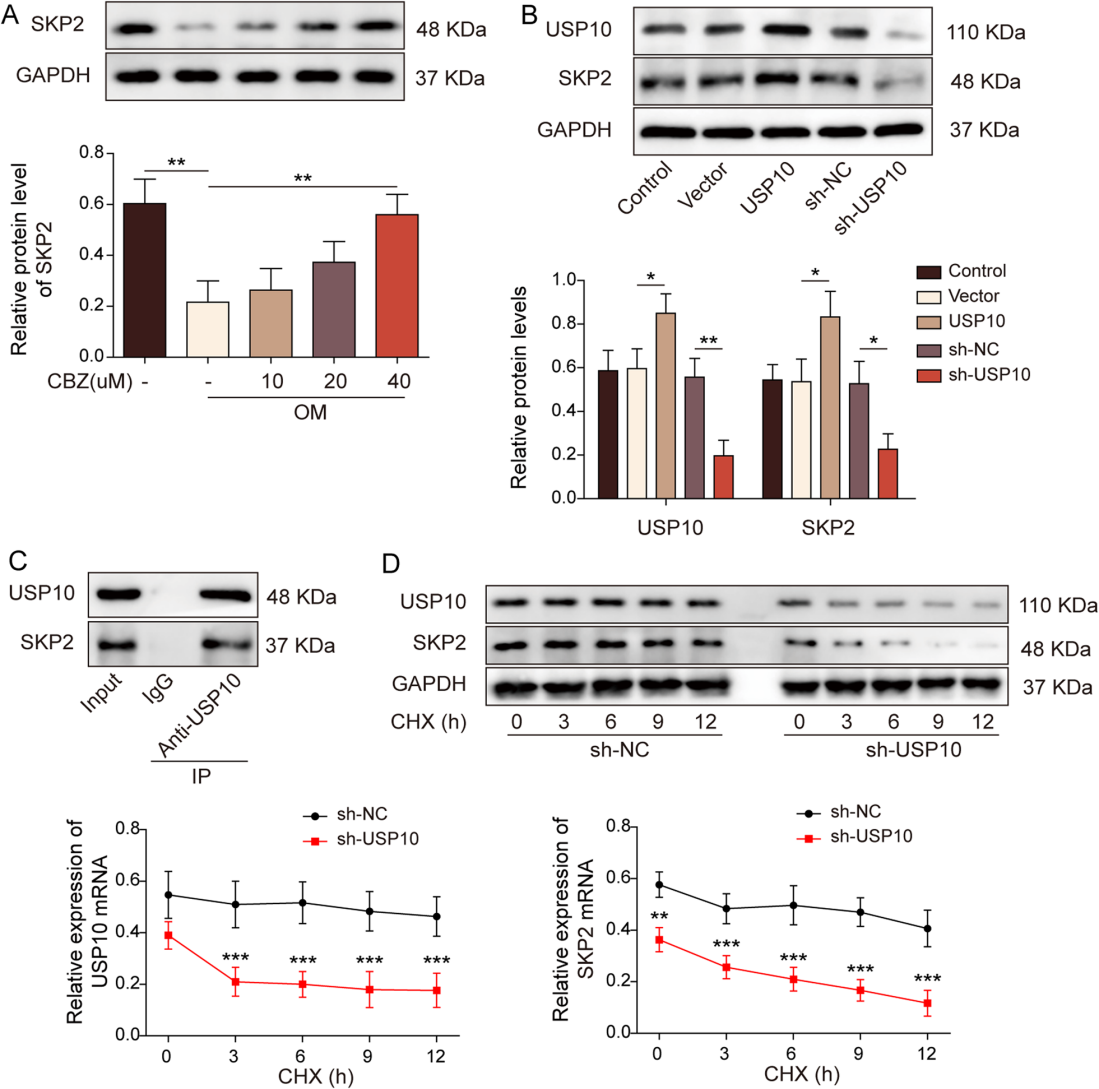
Ubiquitin-specific peptidase 10 increased S-phase kinase-associated protein 2 stability and promoted S-phase kinase-associated protein 2 expression. **A** WB was used to detect SKP2 levels. **B** The USP10 and SKP2 levels were detected by WB. **C** Co-IP was used to verify the combination of USP10 and SKP2. **D** Cycloheximide chase assay verified the effect of the stability of SKP2 protein. The measurement data were presented as mean ± standard error. All data were obtained from at least three replicate experiments. *p < 0.05, ** *p* < 0.01, ***p < 0.001

Next, we examined SKP2 levels in BMSCs treated with CBZ in an osteoblast induction medium OM, the results showed that SKP2 was down-regulated in osteogenic differentiation, while CBZ could enhance SKP2 expression (Fig. 5A). As evidenced in Fig. 5B, the overexpression of USP10 upregulated the level of SKP2, and was reduced by USP10 downregulation. Meanwhile, Co-IP assay verified the interaction between USP10 and SKP2 (Fig. 5C). To detect the effect of USP10 knockdown on the SKP2 protein stability, we treated the cells with cycloheximide. WB results presented that knockdown of USP10 impaired the stability of SKP2 protein in BMSCs (Fig. 5D).

### miR-20a-5p affected the osteogenic effect of CBZ by regulating the USP10/SKP2 axis

**Fig. 6:**
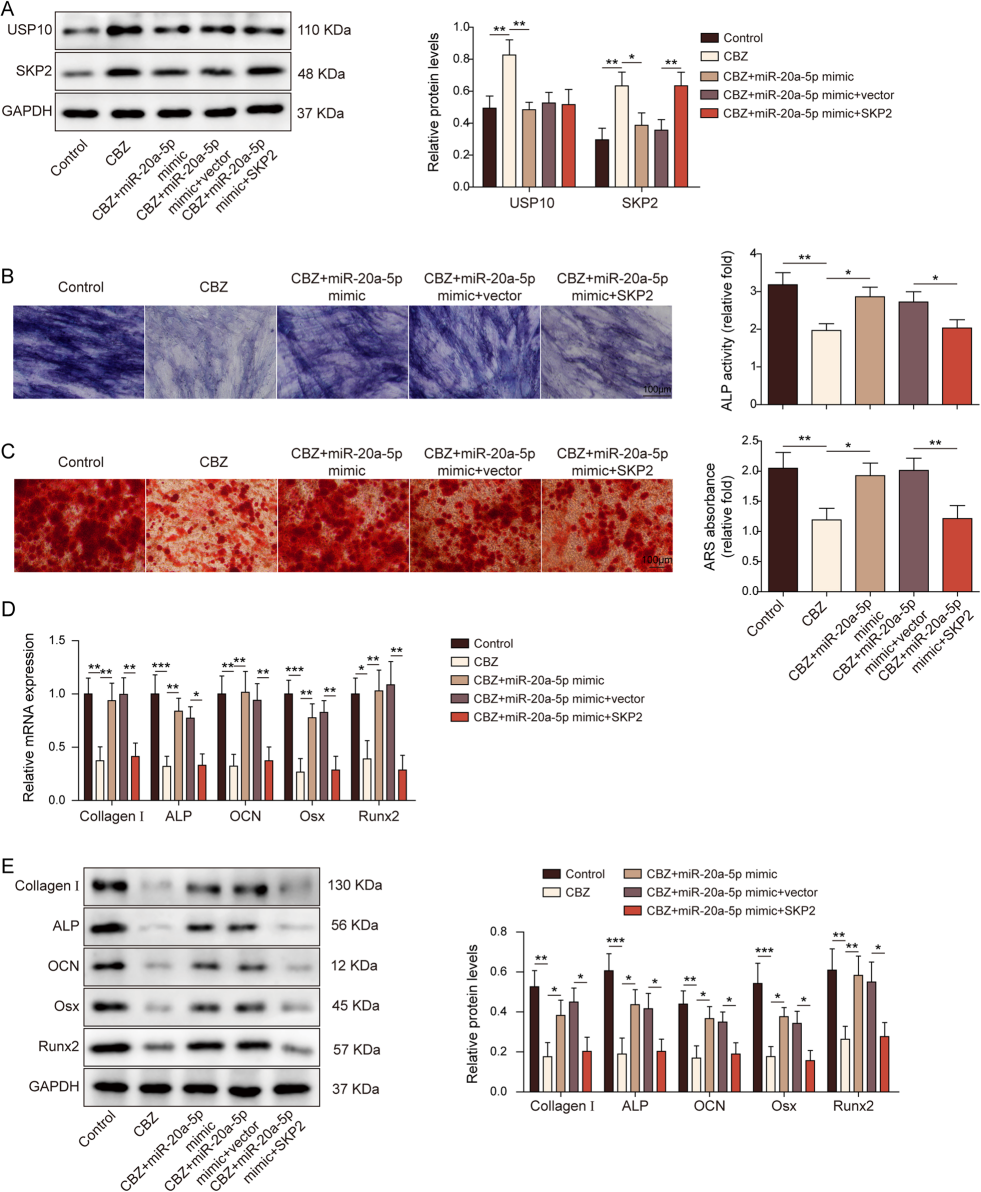
microRNA-20a-5p affected the osteogenic effect of carbamazepine by regulating the ubiquitin-specific peptidase 10/S-phase kinase-associated protein 2 axis. **A** USP10 and SKP2 levels were detected by WB. **B** ALP activity detection. **C** ARS staining was used to detect calcium deposits. Scale bar = 100 µm. The expression of Runx2, ALP, Osx, OCN and Collagen I by RT-qPCR (**D**) and WB (**E**). The measurement data were presented as mean ± standard error. All data were obtained from at least three replicate experiments. *p < 0.05, ** *p* < 0.01, ***p < 0.001

We demonstrated ulteriorly the mechanism of miR-20a-5p in regulating BMSCs osteogenic effect of CBZ. BMSCs were co-transfected with miR-20a-5p mimic and SKP2 overexpression vector. It was proved that USP10 and SKP2 were elevated in the CBZ group compared with the control group. However, overexpression of miR-20a-5p resulted in USP10 and SKP2 decrease, and overexpression of SKP2 could rescue this result (Fig. 6A). ARS staining and ALP assay showed that compared with the control group, the osteogenic differentiation decreased by CBZ or SKP2, while miR-20a-5p promoted osteogenic differentiation (Fig. 6B-C). As displayed in Fig. 6D-E, the mRNA and protein expressions of Runx2, ALP, Osx, OCN and Collagen I in BMSCs treated with CBZ decreased and increased after miR-20a-5p mimic transfection in BMSCs. However, the overexpression of SKP2 could resist the effect of miR-20a-5p.

## Discussion

Osteoporosis is a common metabolic bone disease affected by genetic and environmental factors. It increases the risk of bone fragility and fracture, seriously affecting patients’ quality of life [23, 24]. Maintaining the balance between osteoclast-mediated bone resorption and osteoblast-mediated bone formation is important for the prevention of osteoporosis [25]. At present, the focus of clinical research is still on how to improve osteogenesis or inhibit osteoclast differentiation. In this study, we treated BMSCs with CBZ and constructed cell lines that interfered with or overexpressed miR-20a-5p, USP10 and SKP2 to explore the effects of CBZ by regulating miR-20a-5p/USP10/SKP2 axis on osteoporosis. Our results showed that CBZ reduced miR-20a-5p, alleviated the targeted inhibitory effect of miR-20a-5p on USP10, and further promoted the SKP2 expression, thus inhibiting the BMSCs osteogenic differentiation. Currently, there is no report on the mechanism of CBZ in osteoporosis through the miR-20a-5p/USP10/SKP2 axis, which is first reported in this study.

Recently, miRNAs have been found to regulate gene expression and control osteoblast-dependent bone formation and osteoclast-related bone remodeling [26]. miRNA is a novel regulatory factor that plays a vital role in many cellular processes, including cell differentiation, apoptosis, and bone metabolism, through post-transcriptional regulation of genes [27–29]. miRNAs are increasingly recognized as new therapeutic targets for osteoporosis. For example, Oliviero A et al. reported complex interactions between miRNA and multiple target genes, which may play an important role in gene regulation and homeostasis pathway control in osteoarthritis [30]. Lu X et al. found that miR-15b regulated KDM6B expression by targeting USP7 and inhibited osteoblast differentiation and autophagy, thereby enhancing osteoporosis [31]. CBZ, as AEDs, has been reported to directly affect osteoblast function and possibly bone strength [32]. Feldkamp J et al.’s clinical and experimental data showed that long-term CBZ treatment could decrease bone mineral density. The decreased proliferation of osteocytes observed in the experiment might be related to the damage of new bone formation in clinical practice [33]. Our study found that CBZ inhibited BMSCs osteogenic differentiation by decreasing the mRNA and protein expressions of Runx2, ALP, Osx, OCN and Collagen I. Previous study has shown that miR-20a-5p could promote osteogenic differentiation [34]. However, the molecular mechanism of miR-20a-5p’s involvement in bone formation has not been reported. In this study, CZB reduced the level of miR-20a-5p and thus inhibited the osteogenic differentiation of BMSCs. This is the first time we found that CBZ inhibited osteogenic differentiation of BMSCs by down-regulating miR-20a-5p.

USP10 is reported to be a cytoplasmic ubiquitin-specific protease, and its translocation and stabilization are regulated by ATM-mediated phosphorylation of USP10 at Thr42 and Ser337 [35]. In nonalcoholic fatty liver disease, decreased expression of miR-34a-5p/USP10 was involved in hepatic insulin resistance and steatosis in obese mice. Among them, miR-34a-5p acted as a negative regulator of nonalcoholic fatty liver disease by targeting USP10 [36]. In pancreatic cancer, miR-191 promoted pancreatic cancer development by targeting USP10 [37]. Studies have shown that the possible application of small interfering RNA in the treatment of many diseases caused by altered gene expression function is being increasingly studied [38]. The gene imbalance of inflammatory cytokine small interfering RNA could affect the inflammatory process [39]. In our study of osteoporosis, we found that miR-20a-5p could bond to USP10 and repress its expression. In addition, the knockdown of USP10 could block the osteogenic inhibition induced by CBZ. SKP2 recognized tumor suppressor substrates and was targeted for ubiquitination and degradation by the proteasome via the E3 ligase SKP1-Cullin1-F-box. Among them, SKP2 could be deubiquitinated by USP10 [40]. It was shown that SKP2 ubiquitination was controlled by its E3 ligases (including APC/CFZR1) and deubiquitases (USP10) [41]. In chronic myeloid leukemia cells, USP10 amplified the activation of Bcr-Abl by mediating the deubiquitination of SKP2 and stabilization [22]. In our study, Co-IP and cycloheximide chase assay verified that USP10 increased SKP2 stability and promoted SKP2 expression. Moreover, by co-transfecting miR-20a-5p mimic and/or SKP2 overexpression vector, we found that miR-20a-5p could affect the osteogenic effect of CBZ by regulating USP10/SKP2 axis. Our study provides a theoretical basis for the pathogenesis of osteoporosis and new targets and strategies for the clinical treatment of osteoporosis.

In conclusion, our findings suggested that CBZ regulated USP10 through miR-20a-5p to affect the deubiquitination of SKP2 and inhibit osteogenic differentiation. However, there are still some shortcomings in our study. Due to time and funding constraints, we only conducted in vitro studies on the mechanism of CBZ and miR-20a-5p/USP10/SKP2 axis in osteoporosis. In the future, we will further verify the mechanism of CBZ and miR-20a-5p/USP10/SKP2 axis in osteoporosis in animal models. In addition, our study lacks the clinical effect of CBZ on osteogenic differentiation, which is also the direction of our future efforts.

### Supplementary Information

**Additional file 1**: **Fig. S1**. Detection of IC50 value of carbamazepine on BMSCs. **A** BMSCs were treated with CBZ, cell activity was detected by MTT. The measurement data were presented as mean ± standard error. All data were obtained from at least three replicate experiments. *P < 0.05, **P < 0.01, ***P < 0.001.

## Data Availability

All data generated or analyzed during this study are included in this published article.

## Abbreviation

AEDs: Antiepileptic drugs
CBZ: Carbamazepine
SKP2: S-phase kinase-associated protein 2
BAMBI: Bone morphogenetic protein and activin membrane-bound inhibitor
KDM5B: Lysine demethylase 5B
BMSCs: Bone marrow mesenchymal stem cells
FBS: Fetal bovine serum
RT-qPCR: Reverse transcriptase-quantitative polymerase chain reaction
WB: Western blot
ARS: Alizarin Red S
ALP: Alkaline phosphatase
WT: Wild type
MUT: Mutant
RIP: RNA immunoprecipitation
Co-IP: Co-Immunopurification
ANOVA: Analysis of variance
