# Supplementary figures and images for: Carbamazepine regulates USP10 through miR-20a-5p to affect the deubiquitination of SKP2 and inhibit osteogenic differentiation
Source: J Orthop Surg Res. 2023 Nov 1;18:820. doi: 10.1186/s13018-023-04169-7 (PMC10619296; doi:10.1186/s13018-023-04169-7)

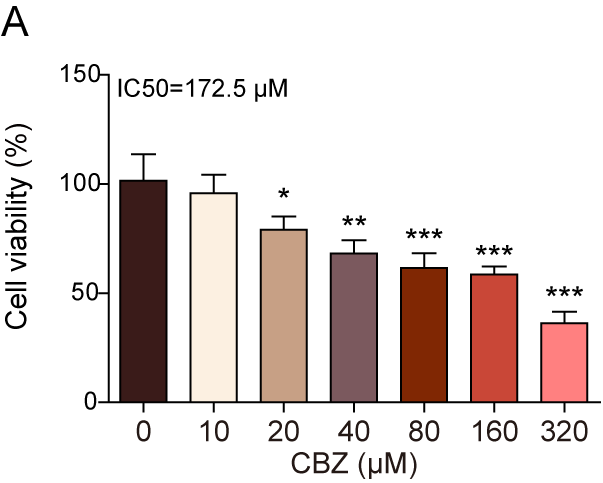

Supplement: Supplementary file 1 — Additional file 1: Fig. S1. Detection of IC50 value of carbamazepine on BMSCs. A BMSCs were treated with CBZ, cell activity was detected by MTT. The measurement data were presented as mean ± standard error. All data were obtained from at least three replicate experiments. *P < 0.05, **P < 0.01, ***P < 0.001. [file 13018_2023_4169_MOESM1_ESM.tif]
